# The projected impact of the COVID-19 lockdown on breast cancer deaths in England due to the cessation of population screening: a national estimation

**DOI:** 10.1038/s41416-022-01714-9

**Published:** 2022-02-02

**Authors:** Stephen W. Duffy, Farah Seedat, Olive Kearins, Mike Press, Jackie Walton, Jonathan Myles, Daniel Vulkan, Nisha Sharma, Anne Mackie

**Affiliations:** 1 Wolfson Institute of Population Health, Queen Mary University of London, Charterhouse Square, London, EC1M 6BQ UK; 2 UK National Screening Committee, Office for Health Improvement and Disparities, Department of Health and Social Care, 39 Victoria Street, London, SW1H 0EU UK; 3 London School of Hygiene and Tropical Medicine, Keppel St, Bloomsbury, London, WC1E 7HT UK; 4 Public Health England, Screening Division, Floor 5, Wellington House, 133-155 Waterloo Road, London, SE1 8UG UK; 5 Breast Unit, Level 1 Chancellor Wing, St James Hospital, Beckett Street, Leeds, West Yorkshire LS9 7TF UK

**Keywords:** Cancer screening, Viral infection, Epidemiology, Population screening

## Abstract

**Background:**

Population breast screening services in England were suspended in March 2020 due to the COVID-19 pandemic. Here, we estimate the number of breast cancers whose detection may be delayed because of the suspension, and the potential impact on cancer deaths over 10 years.

**Methods:**

We estimated the number and length of screening delays from observed NHS Breast Screening System data. We then estimated additional breast cancer deaths from three routes: asymptomatic tumours progressing to symptomatically diagnosed disease, invasive tumours which remain screen-detected but at a later date, and ductal carcinoma in situ (DCIS) progressing to invasive disease by detection. We took progression rates, prognostic characteristics, and survival rates from published sources.

**Results:**

We estimated that 1,489,237 women had screening delayed by around 2–7 months between July 2020 and June 2021, leaving 745,277 outstanding screens. Depending on how quickly this backlog is cleared, around 2500–4100 cancers would shift from screen-detected to symptomatic cancers, resulting in 148–452 additional breast cancer deaths. There would be an additional 164–222 screen-detected tumour deaths, and 71–97 deaths from DCIS that progresses to invasive cancer.

**Conclusions:**

An estimated 148–687 additional breast cancer deaths may occur as a result of the pandemic-related disruptions. The impact depends on how quickly screening services catch up with delays.

## Background

The Coronavirus disease 2019 (COVID-19) pandemic resulted in the reconfiguration of health service provision in England and worldwide [1, 2]. Health services were re-purposed, clinicians redeployed, and non-emergency treatments delayed. Since the national lockdown was announced in the UK on 23 March 2020, social distancing measures were applied, vulnerable people were encouraged to shield, and declines in attendance at healthcare settings were reported. The breast screening programme is no exception.

It has been found that breast screening prevents substantial numbers of breast cancer deaths [3, 4]. In England, the breast screening programme offers mammography every 3 years to women aged 50 to less than 71 years. Women with abnormal screening results are recalled for assessment (clinical examination, imaging ± biopsy). The programme screens more than two million women annually and is estimated to prevent 1300 UK deaths annually [3, 5].

Between 17 and 31 March 2020, all 78 breast screening services in England suspended invitations for screening. Efforts were focussed on retaining the screening pathways for women at high familial risk and those with existing screen-positive findings. While restoration of population breast cancer screening is underway (all services resumed invitations by end of September 2020), social distancing, PPE and infection control requirements prevent services from re-establishing previous capacity. It is currently unclear when or if services will return to a pre-COVID model and recover timely invitations.

Maringe et al. estimated the effects of the pandemic on cancer deaths based on the number of cancers whose detection would shift from either a screening or routine referral pathway to an urgent referral pathway (2-week wait referrals and emergency presentations), which are associated with later stage at diagnosis and therefore lower survival [6]. They assumed 25% of cancers shifted to urgent pathways, and estimated that between 266 and 358 additional deaths would accrue in 5 years as a result of the shifting of screen detected cancers and routine symptomatic referrals combined. There were no detailed data specifically on screens delayed or missed.

In this study, we focus specifically on screening. We estimate the number of breast cancers whose detection may be delayed as a result of the suspension of population screening during the first lockdown in England, using detailed information on screens delayed, tumour progression and survival. From these, we estimate the potential impact on breast cancer deaths over 10 years.

## Methods

### Primary analysis: effect of shifting from asymptomatic to symptomatic status

A major effect of the screening cessation would be the progression of asymptomatic tumours to symptomatic disease, which would now be diagnosed symptomatically instead of being screen-detected. This shift would result in changes in the prognostic status and 10-year survival of the women diagnosed. We first estimated the number of delayed screens and corresponding lengths of the delays. From these, we estimated the number of invasive cancers which would have been screen-detected in a typical year, but which are likely to arise as symptomatic cancers because of the COVID-related pause in screening, and the reduced coverage during the catch-up period. We then estimated the additional breast cancer deaths likely to occur within 10 years as a result of this shift from screen-detected to symptomatic disease using the average Nottingham Prognostic Index (NPI) of screen-detected and interval cancers [7].

We assumed that since we were estimating results for those who would have attended screening if invited, their NPI if diagnosed symptomatically would be similar to that of interval cancers rather than of non-attenders for screening. Symptomatic cancers in screening attenders tend to have more favourable prognostic characteristics than symptomatic cancers in non-attenders.

### Further analyses: effect of later diagnosis but still by screening, and progression of ductal carcinoma in situ (DCIS)

We carried out two further analyses. One analysis estimated the effect on outcome of delayed diagnosis in cancers that were nevertheless screen detected, albeit at a later date than their scheduled screen. The second analysis was to estimate the effect on outcome of delayed diagnosis in cancers that would have been diagnosed as DCIS at the time of the scheduled screen.

**Fig. 1:**
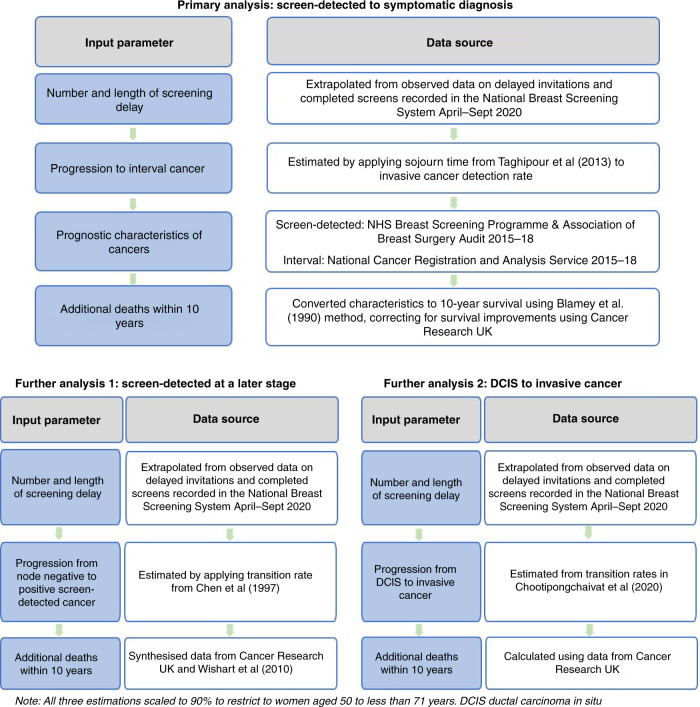
Structure and data sources. Primary analysis, screen-detected to symptomatic diagnosis; further analysis 1, screen-detected at a later stage; and further analysis 2, DCIS to invasive cancer.

Figure 1 shows the overall structure of the estimations from screening cessation to additional deaths through the three routes described above. The analysis section below describes in more detail the methods used to obtain the estimates.

### Data sources

Figure 1 shows the data sources used in the three estimations. The population of interest was the English breast cancer screening cohort of women aged 50 to less than 71 years who were eligible for screening during cessation and recovery. To estimate the number of women who would have had their screening delayed and the length of the delay, we used data from the NHS Breast Screening System (NBSS) as a starting point. The prognostic characteristics of screen-detected cancers (see Table 1) were collected from the NHS Breast Screening Programme and Association of Breast Surgery (NHSBSP, ABS) audit for 3 financial years 2015–18 [8]. The characteristics for interval cancers were collected from the National Cancer Registration and Analysis Service (NCRAS) for 4 calendar years 2015–18 (see Table 1) [9]. The remaining parameters were taken from the literature but adjusted as described below.

**Table 1:** Size, node status and grade of screen-detected and interval cancers, England, NHS Breast Screening Programme, women aged 47/50 or over.

Factor	Category/aspect	Screen-detected cancers, April 2015–March 2018 (3 years)	Interval cancers, 2015–18 (4 years)
Size (invasive only)	0 to ≤20 mm	30,046	9237
	>20 to ≤50 mm	8270	9966
	>50 mm	834	1673
	Mean	16.4	25.0
	Standard Deviation (SD)	12.2	17.2
	Number unknown	1602	5246
Positive nodes (invasive only)	0	31,968	12,316
	1–3	6275	4862
	4+	1484	1636
	Unknown	1025	7308
Grade (invasive only)	1	9941	2442
	2	22,729	13,134
	3	7799	9599
	Unknown	283	947
NPI (invasive only)	Mean	3.5	4.2
	Standard deviation (SD)	1.0	1.1
	Median	3.3	4.3
	Quartile 1	3.1	3.3
	Quartile 3	4.2	4.7
	Number unknown	1824	7044
	% unknown	4.5%	27.0%

### Statistical analysis

The methods to estimate the number and length of delays are described in the Supplementary material and are based on observed numbers of delayed invitations and actual screens carried out by month in data available from the NBSS, as of December 2020 (see Supplementary Tables A1-A3).

#### Primary analysis: effect of shifting from asymptomatic to symptomatic disease

We estimated the proportion of cancers moved to symptomatic diagnosis as a result of the pause using Taghipour et al.’s estimate of progression from asymptomatic to symptomatic invasive breast cancer [10]. They estimate mean sojourn time as 3 years, corresponding to a transition rate from presymptomatic to symptomatic disease of 1/3 = 0.3333 per year. This would mean, for example, that if a tumour that would have been screen-detected had the scheduled screen delayed by 6 months = 0.5 years, the probability that it would become symptomatic in those 6 months would be estimated as

$$P = {\int}_0^{0.5} {0.3333e^{ - 0.3333 \times t}dt = 1 - e^{ - 0.5 \times 0.3333} = 0.15}$$

We estimated the numbers of invasive tumours shifted based on an invasive cancer detection rate of 6.9 per thousand screened [5]. Thus, for example, if 40,000 screens take place in a given month, and those screens were delayed by 6 months, the estimated number of invasive cancers shifted from screen-detected to symptomatic is

$$N = 0.15 \times 40 \times 6.9 = 41$$

Conversion of NPI to 10-year survival used the method of Blamey et al. [7], which estimated the dependence of 10-year net survival on NPI in 2238 cancers diagnosed in 1990–99.

#### Missing data

Table 1 shows the distributions of size, node status, histological grade and NPI of screen-detected and interval cancers from 2015–18. Note that 4.5% of screen-detected cancers and 27.0% of interval cancers had missing NPI. We carried out two analyses, one optimistic, assuming missing at random, and one pessimistic, assuming that those with missing NPI have the same survival as those with missing stage, since these are likely to be largely the same populations. Survival data was sourced from the Cancer Research UK website, however, it does not give 10-year survival for those with missing stage but quotes 5-year survival as 69.1% [11]. From the same source, overall 5-year survival and 10-year survival were given as 85.0% and 75.9%, respectively, a ratio of 0.893. Applying this to the 69.1%, we estimated 10-year survival in those with missing NPI as 61.7%.

#### Correcting for time trends in survival

Blamey et al. used survival from cancers diagnosed in 1990–99 and since this work predicts survival for cancers diagnosed in 2020 and 2021, we corrected for time trends in survival [7]. On the Cancer Research UK website, 10-year survival figures are given by calendar period of diagnosis, as in Table 2 [11]. We performed regression of the log odds of survival on time of diagnosis and extrapolated to the year 2020. This gave an estimated odds of 10-year survival for 2020 as 6.03, corresponding to 86% survival for tumours diagnosed in 2020, compared to the odds of 4.00 (80% survival) for cancers diagnosed in 1990–99 in Blamey et al. [7]. We, therefore, inflated the odds of survival from Blamey et al. for screen-detected and symptomatic cancers with known NPI by 6.03/4.00.

**Table 2:** Ten-year survival from breast cancer by time of diagnosis.

Years of diagnosis	Ten-year survival
1971–1972	40.0
1980–1981	48.4
1990–1991	60.0
2000–2001	71.5
2005–2006	75.6
2010–2011	78.4

#### Further analyses: delayed screen detection and DCIS

To estimate the effect of delayed diagnosis within cancers which remain screen-detected, we estimated the likely effect of the delay on lymph node status, combined with the survival difference between node positive and node negative cancers. From Chen et al. [12], the transition rate from node negative to node positive breast cancers is estimated at ~0.19. We combined this with the delay in diagnosis among the screen-detected cancers and the proportion of screen-detected cancers node negative to obtain the number of screen detected cancers which would have been node negative at diagnosis at the original scheduled screen date, but are in fact node positive at the actual screen date. Cancer Research UK gives 10-year net survival for all breast cancers as 76% [11]. From Wishart et al., the estimated relative hazard for node positive compared to node negative cancers was 3.18 (by weighted average of the two categories of node positive) [13]. Synthesising these gave 10-year survival estimates of 85% for node negative cancers and 61% for node positive. From these we calculated the additional deaths from delayed diagnosis within cancers which remained screen-detected.

For DCIS, we first assumed 100% 10-year survival of screen-detected in situ cancer, and screen detection rates of DCIS as 1.4 per thousand [5]. Chootipongchaivat et al. find a range of proportions of DCIS with progressive potential [14]. We assumed the midpoint, 76%, so we multiplied the number of expected DCIS cases by 0.76 to obtain the number with progressive potential. The same paper estimates a range of times to progression to invasive cancer in the progressive cases, and again we took the midpoint, 1.35 years, giving a transition rate from in situ to invasive of 1/1.35 = 0.74.

From this, we estimated the probability of becoming invasive in 4.5 months (see Supplementary material), and therefore the number of cancers that would have been in situ screen-detected but will now be invasive. Assuming they will all be diagnosed at invasive stage 1, we compared the stage 1 ten-year survival, estimated from the 5-year survival for stage 1 cancers of 0.98 multiplied by the ratio of 10-year to 5-year survival of 0.89 [11], giving 87% survival, with the 100% assumed above, to obtain the additional deaths.

### Results for the age group 50 to less than 71

Figures available on delayed invitations and screens taking place pertained to screening at all ages. In the year 2018–19, screens taking place and cancers detected in women aged 50–<71 constituted 83% of all screens and cancers detected. It is anticipated that since the 2020 lockdown and the cessation of screening in relation to the AgeX trial [15], which is evaluating extending the screening age range to a lower limit of 47 years and an upper limit of 73, the figure would be close to 90%. We, therefore, scaled down our outcomes by a factor of 0.9 to obtain approximate estimates for the 50–<71 age group.

## Results

### Primary analysis

#### Number and lengths of delays to screens, and consequent shift to symptomatic disease

Table 3 shows the estimated numbers of screens, months by which they were delayed, and from these, the cancers shifted from screen-detected to symptomatic status for each month from July 2020 to June 2021. There was negligible screening activity between April and June 2020, rising every month thereafter and stabilising at just over 142,000 (see Supplementary Table A1 and Supplementary Table A2). The tendency is for the length of the delay to rise but it can fall between months depending on the average delay in those remaining unscreened at the end of the previous month (see Supplementary Table A3). We estimated that 1200 cancers shifted from screen-detected to symptomatic as a result of the delays to these 1,489,237 screens.

**Table 3:** Estimated numbers of screens by month with average delays and consequent numbers of cancers shifted from screen-detected to symptomatic status.

Month	Screens in that month	Average delay (months) of screens in that month	Number of cancers shifted from screen-detected to symptomatic
July 2020	33,547	2.75	17
August 2020	73,123	2.33	32
September 2020	114,277	2.42	51
October 2020	133,199	2.68	66
November 2020	139,690	3.45	88
December 2020	141,601	4.28	110
January 2021	142,140	4.17	107
February 2021	142,290	4.65	119
March 2021	142,332	5.31	135
April 2021	142,344	6.31	158
May 2021	142,347	7.31	180
June 2021	142,348	5.41	137
Total	1,489,237	–	1200

In a typical year, we would expect 2,234,514 screens. Thus, we estimate that an additional 745,277 screens, which should take place between July 2020 and June 2021 will not do so. We assume that this population is screened within the year following the end of June 2021, with an average delay of 13.25 months (see Supplementary material). We, therefore, estimate that a further 1583 cancers have been or will be shifted from screen-detected to symptomatic status, giving a total of 2783 cancers. If, more pessimistically, we assume that half of the backlog is never caught up with, which would mean 50% of the outstanding population of 745,277 never receives a screen, and therefore all cancers occurring in this 50% are diagnosed symptomatically, the total number of cases shifted to symptomatic would be 1200 + 3363 = 4563 cancers.

#### Ten-year survival difference between screen-detected and symptomatic cancers

For our optimistic first estimate (missing at random), we estimate expected 10-year survival in screen-detected and interval cancers based on the NPI, using the survival observed for cancers diagnosed in 1990–99 in Blamey et al. [7]. The latter gives 10-year percentage survival as

$${{{{{\rm{S}}}}}} = - 1.62 \times {{{{{\rm{NPI}}}}}}^2 + 1.25 \times {{{{{\rm{NPI}}}}}} + 102.77$$

From the NCRAS and screening audit data, for those with known NPI, the average for screen-detected cancers was 3.5 and for interval cancers 4.2 (Table 1). These would give average 10-year survival figures of 87.3% for screen-detected cancers and 79.4% for symptomatic cancers.

This suggests that, of those cancers which would have been screen-detected, but which were symptomatically diagnosed due to the hiatus, an additional 7.9% (87.3%–79.4%) would die of the disease in 10 years as a consequence.

From the estimated 10-year survival of 61.7% for those with missing NPI, and using the percentages missing (4.5% in screen-detected cancers, 27.0% in interval cancers), we estimated 10-year survival in screen-detected cancers as

$${{{{{\rm{SD}}}}}} = 0.955 \times 87.3 + 0.045 \times 61.7 = 86.1{{{{{{{\mathrm{\% }}}}}}}}$$

for symptomatic cancers, the estimate was

$${{{{{\rm{SY}}}}}} = 0.730 \times 79.4 + 0.270 \times 61.7 = 74.6{{{{{{{\mathrm{\% }}}}}}}}$$

Thus, more pessimistically assuming those with missing NPI have same survival as those missing stage, we estimated that of those whose detection mode has been shifted from screening to symptomatic, an additional 11.5% would die from breast cancer in 10 years.

#### Correcting for time trends in survival

Inflating the odds of survival as described above gave expected 10-year survival of 91.2% for screen-detected and 85.3% for interval cancers under the optimistic missing at random scenario. For the more pessimistic scenario assuming that those with missing NPI have the same survival as those with missing stage, we have

$${{{{{\rm{SD}}}}}} = 0.955 \times 91.2 + 0.045 \times 61.7 = 89.9{{{{{{{\mathrm{\% }}}}}}}}$$

for screen-detected cancers and

$${{{{{\rm{SY}}}}}} = 0.730 \times 85.3 + 0.270 \times 61.7 = 78.9{{{{{{{\mathrm{\% }}}}}}}}$$

for symptomatic cancers. We did not inflate the survival of those with missing NPI since this was derived from contemporaneous data [5].

The above suggests that of those whose detection mode was shifted from screening to symptomatic, between 5.9% (91.2%–85.3%) and 11.0% (89.9%–78.9%) would additionally die of breast cancer.

#### Predicted additional numbers of deaths as a result of a shift to symptomatic disease

We estimated above that 2783 cancers in England would be shifted from screen-detected to symptomatic disease as a result of the disruption to the programme. Converting these to additional breast cancer deaths in the optimistic scenario for missing data would imply that an additional 164 breast cancer deaths would occur. In the more pessimistic scenario, there would be an additional 306 breast cancer deaths.

With the assumption that half of the backlog of 745,277 delayed screens remains at the end of June 2021, there would be 4,563 cancers shifted from screen-detected to symptomatic, with an estimated number of additional breast cancer deaths of between 269 and 502 depending on the assumption with respect to missing NPI.

For the age group 50–<71, we scaled the results down by a factor of 0.9 as noted above. This would give, under the assumption that the remaining backlog at the end of June 2021 would be screened within a year, a total of 2505 cancers in the age group 50–<71 shifted from screen-detected to symptomatic. This would give a range of 148–275 additional breast cancer deaths, depending on the assumption about unknown NPI. Assuming that half of the backlog would not eventually be screened, we would expect 4107 cancers shifted to symptomatic status, with a range of 242–452 additional breast cancer deaths.

### Later diagnosis of screen-detected cancers and progression of DCIS

The number of extra deaths from screen detected cancers in the 50–<71 age group whose diagnosis was delayed by 4.5 months on average, was calculated from the 1,489,237 delayed screens as shown in Table 4. The estimated number of additional deaths was 106.

**Table 4:** Calculation of additional deaths from delayed diagnosis of screen detected cancers which remain screen-detected.

Quantity	Calculation
Expected cancer diagnoses	0.0069 × 1,489,237 = 10,276
Removal of cancers diagnosed symptomatically	10,276 – 1200 = 9076
Proportion node negative from Table 1	31,968/(31,968 + 6275 + 1484 + 1025) = 0.7845
Number node negative	0.7845 × 9076 = 7120
Proportion progressing to node positive in 4.5 months	1-(exp(−0.19 × 4.5/12)) = 0.069
Number of additional node positive cancers	0.069 × 7120 = 491
Additional fatality over 10 years for node positive	24%
Additional deaths over 10 years	491 × 0.24 = 118
Scaled down by 0.9 for age group 50–<71	0.9 × 118 = 106

Whilst any clearing of the backlog will have the effect of reducing the number of women who will be diagnosed following symptomatic presentation, some of the women whose cancers are now screen-detected will nevertheless be diagnosed later than they would have been if there had not been a cessation at all. We estimated that, if the entire backlog is caught up with, 536 of these women would have their diagnosis delayed sufficiently for their cancers to be node-positive rather than node-negative, resulting in 116 additional deaths in the 50–<71 age group. If 50% of the backlog is caught up with, 268 women would be in this position, with 58 additional deaths.

Table 5 shows the estimation of additional deaths from progression of DCIS in the 1,489,237 delayed screens taking place from July 2020 to June 2021. We estimated that 45 additional deaths would result from progression of DCIS to invasive disease. Similar calculations give 52 deaths from progression of DCIS in the 745,277 delayed screens outstanding at the end of June 2021 if the entire backlog is caught up with, and 26 if 50% is caught up with.

**Table 5:** Calculation of additional deaths from progression of DCIS.

Quantity	Calculation
Expected DCIS diagnoses	0.0014 × 1,489,237 = 2085
Potentially progressive DCIS cases	0.76 × 2085 = 1585
Proportion progressing to invasive cancer	1-(exp(−0.74 × 4.5/12)) = 0.24
Number progressing to invasive cancer	0.24 × 1585 = 384
Additional breast cancer deaths as a result	0.13 × 384 = 50
Scaled down by 0.9 for age group 50–<71	0.9 × 50 = 45

Table 6 shows additional deaths for all combinations of the four assumptions, with respect to missing NPI, delayed diagnosis of screen-detected cancers, delayed diagnosis of DCIS and proportion of the remaining backlog at June 2021 who eventually are screened. The range of possible additional deaths extends from 148 to 687.

**Table 6:** Projected additional breast cancer deaths incurred due to the 2020 cessation of screening, for combinations of assumptions with respect to missing data and the remaining backlog at the end of June 2021.

	Whole of backlog cleared	50% of backlog cleared
Source of additional breast cancer deaths		
- Symptomatic detection of cancers that would have been screen-detected^a^	148–275	242–452
- Delayed screen detection	222	164
- Progression of DCIS	97	71
Total^a^	467–594	477–687

^a^Range represents optimistic and pessimistic scenarios.

## Discussion

From published data, we calculated a range of likely extra breast cancer deaths over 10 years as a result of the screening suspension in 2020 and the anticipated rate of ‘catch-up’ of the programme. Depending on assumptions, we predicted that between 148 and 687 additional breast cancer deaths would occur. While this range is wide, the assumptions pertaining to the lower estimates, such as missingness at random and no effect of delays in breast cancers diagnosed later but still by screening, are optimistic. It is likely that the true numbers are closer to the upper end of the range.

Maringe et al. estimated an additional 266–358 deaths within 5 years as a result of screening and symptomatic delays from the pandemic in England [6]. These figures for 5 years for both screening and symptomatic delays are consistent with our figures for 10 years for screening alone. By contrast, SAGE estimated 1–20 excess deaths in 5 years from a 6 months screening cession in England [16]. However, the latter assumed very low estimates of stage progression (1%, 2%, and 4% over a year), which are not supported by the literature [12]. In addition, the SAGE model did not consider the longer impact of the length of recovery. We have attempted to account for this recovery phase in the current estimation. Our estimates are more in line with the results from the independent reviews, which estimated that the programme prevents between 1300 and 1700 deaths per annum [17, 18].

The estimated impact of the breast screening disruption on mortality from formal population models varies internationally for many reasons, including different screening strategies [19, 20]. In line with our estimations, a 3-month disruption in Scotland was estimated to result in 6.3% additional deaths while a 6 month disruption would result in 22.3% [19]. Likewise, in Canada, it was estimated that a 3-month disruption could result in 310 extra advanced cancers and 110 additional deaths in 2020–2029 [21]. In Australia, a very small reduction in 5-year survival was estimated for moving from a 3 month to a 12 month pause (90.4%–89.5%) [22]. However, this model assumed very different policies of populations prioritised for screening and a rapid return to full capacity after a pause. Also, in Australia, the screening interval is either annual or biennial whereas in the UK it is triennial, therefore, a delay of 12 months in Australia gives the standard interval in the UK.

To our knowledge, this is one of the few studies in England to model the impact of the COVID-19 suspension to the breast screening programme on breast cancer mortality. We used real life data on screening activity and numbers delayed from the national screening programme to inform our estimates. Where possible, we used published data for estimation. However, there are limitations. We estimated effects of the three routes to additional cancer deaths independently. We did not develop a single comprehensive model of tumour progression as we wished to obtain estimates in a timely and transparent manner. We did not explicitly model lead time in our survival figures for screen detected cancers. However, our predicted deaths are based not on whether the tumour was screen detected or symptomatic per se, but on invasive status, lymph node status or NPI. Finally, our numbers of delayed screens and the rate of recovery are estimates dependent on assumptions. However, since our initial calculations, the observed number screened in November 2020 has become available, and is 146,827, close to the 139,690 estimated. Other countries may wish to replicate the methods used here for a timely and transparent estimate of screening suspension for their context.

The suspension and recovery of screening services will result in delays in diagnosis, which may have a substantial negative impact on women’s survival for the next 10 years. There is the additional impact of delays in diagnostic and treatment pathways thereafter that may increase the number of excess deaths [23–25]. To minimise the excess deaths, screening services need to overcome current challenges and increase their capacity to catch up with the backlog. Services have been directed to prioritise cancelled screens first and then invite delayed screens. The programme has moved to open appointments rather than timed to maximise utilisation of appointments. Additional options are to extend hours or days of operation, but this implies additional staff and equipment. Concurrently, it is important to remind women of the importance of attending for screening and reassuring them that all the safety measures are in place.

While our estimates are at national population level, there will be differences across England. We could not account for the impact in different populations. We also do not know the impact of open invitations on different groups. Estimating the impact of the screening cessation on health inequalities and on excess mortality within vulnerable subgroups is critical to explore and if necessary, to mitigate.

To conclude, we estimated that the suspension and recovery of breast screening services in England may result in a substantial number of excess deaths from breast cancer in the next 10 years. The impact is dependent on how quickly services catch up. We will, in future, come to appreciate the impact of the delays. For the current time, the backlog needs to be addressed as quickly as possible by increasing screening capacity and ensuring public health messaging encourages women to attend their open screening invitations.

## Supplementary information

Supplementary material

## Data Availability

We did not collect novel data for this study. The data we used for our estimation input have been described in the text and tables and have been referenced for readers.

## Code availability

Computer code used for calculations (in STATA version 15) is obtainable from SWD.

## References

[1] Smithard DG, Haslam J (2021). COVID-19 pandemic healthcare resource allocation, age and frailty. New Bioeth.

[2] Supady A, Curtis JR, Abrams D, Lorusso R, Bein T, Boldt J (2021). Allocating scarce intensive care resources during the COVID-19 pandemic: practical challenges to theoretical frameworks. Lancet Respir Med.

[3] The benefits and harms of breast cancer screening: an independent review. The Lancet. 2012;380:1778-86. 10.1016/S0140-6736(12)61611-0 23117178

[4] Schünemann HJ, Lerda D, Quinn C, Follmann M, Alonso-Coello P, Rossi PG (2020). Breast cancer screening and diagnosis: a synopsis of the european breast guidelines. Ann Intern Med.

[5] Screening and Immunisations Team, NHS Digital. Breast Screening Programme: England 2018-19. Leeds: NHS Digital; 2020.

[6] Maringe C, Spicer J, Morris M, Purushotham A, Nolte E, Sullivan R, et al. The impact of the COVID-19 pandemic on cancer deaths due to delays in diagnosis in England, UK: a national, population-based, modelling study. Lancet Oncol. 2020;21:1023-34. 10.1016/S1470-2045(20)30388-0 PMC7417808 32702310

[7] Blamey RW, Ellis IO, Pinder SE, Lee AH, Macmillan RD, Morgan DA (2007). Survival of invasive breast cancer according to the Nottingham Prognostic Index in cases diagnosed in 1990–1999. Eur J Cancer (Oxf, Engl: 1990).

[8] NHS Breast Screening Programme and Association of Breast Surgery Screening Audit Group. An audit of screen detected breast cancers for the year of screening April 2018 to March 2019 England Public Health England, Association of Breast Surgery; 2020.

[9] Public Health England. National Cancer Registration and Analysis Service (NCRAS) England: Public Health England; 2020; https://www.gov.uk/guidance/national-cancer-registration-and-analysis-service-ncras.

[10] Taghipour S, Banjevic D, Miller AB, Montgomery N, Jardine AK, Harvey BJ (2013). Parameter estimates for invasive breast cancer progression in the Canadian National Breast Screening Study. Br J Cancer.

[11] Cancer Research UK. Breast cancer survival statistics. https://www.cancerresearchuk.org/health-professional/cancer-statistics/statistics-by-cancer-type/breast-cancer/survival. Last accessed 1st June 2021.

[12] Chen H, Duffy S, Tabar L, Day N (1997). Markov chain models for progression of breast cancer. Part I: tumour attributes and the preclinical screen-detectable phase. J Epidemiol Biostat..

[13] Wishart G, Greenberg D, Chou P, Brown C, Duffy S, Purushotham A (2010). Treatment and survival in breast cancer in the Eastern Region of England. J Ann Oncol.

[14] Chootipongchaivat S, van Ravesteyn NT, Li X, Huang H, Weedon-Fekjær H, Ryser MD (2020). Modeling the natural history of ductal carcinoma in situ based on population data. Breast Cancer Res.

[15] Cancer Research UK. A trial to evaluate an age extension to the NHS Breast Screening Programme 2020; https://www.cancerresearchuk.org/about-cancer/find-a-clinical-trial/a-study-to-evaluate-an-age-extension-of-the-nhs-breast-screening-programme.

[16] Department of Health and Social Care, Office for National Statistics, Government Actuary’s Department, Home Office. Direct and Indirect Impacts of COVID-19 on Excess Deaths and Morbidity. 2020.

[17] Marmot MG, Altman DG, Cameron DA, Dewar JA, Thompson SG, Wilcox M (2013). The benefits and harms of breast cancer screening: an independent review. Br J cancer.

[18] Richards M. The independent review of adult screening programmes in England. England: NHS; 2019.

[19] Breast Screening Working Group (WG2) of the Covid-19 and Cancer Global Modelling Consortium. The impact of the Covid-19 pandemic on breast cancer early detection and screening. Prev Med. 151:106585. 10.1016/j.ypmed.2021.106585 PMC8241687 34217412

[20] Kregting LM, Kaljouw S, de Jonge L, Jansen EEL, Peterse EFP, Heijnsdijk EAM, et al. Effects of cancer screening restart strategies after COVID-19 disruption. Br J Cancer. 2021;124:1516–23. 10.1038/s41416-021-01261-9 PMC7957464 33723386

[21] Yong JH, Mainprize JG, Yaffe MJ, Ruan Y, Poirier AE, Coldman A, et al. The impact of episodic screening interruption: COVID-19 and population-based cancer screening in Canada. J Med Screen. 2020:28:100-107. 10.1177/0969141320974711 PMC7691762 33241760

[22] Nickson C, Procopio P, Deij S, Velentzis L. COVID-19 scenario modelling for cancer screening programs, the BreastScreen Australia Program. In: Division CR, editor. New South Wales: Cancer Council NSW; 2020.

[23] Sud A, Torr B, Jones ME, Broggio J, Scott S, Loveday C (2020). Effect of delays in the 2-week-wait cancer referral pathway during the COVID-19 pandemic on cancer survival in the UK: a modelling study. Lancet Oncol.

[24] Lai AG, Pasea L, Banerjee A, Hall G, Denaxas S, Chang WH (2020). Estimated impact of the COVID-19 pandemic on cancer services and excess 1-year mortality in people with cancer and multimorbidity: near real-time data on cancer care, cancer deaths and a population-based cohort study. BMJ Open.

[25] Hanna TP, King WD, Thibodeau S, Jalink M, Paulin GA, Harvey-Jones E, et al. Mortality due to cancer treatment delay: systematic review and meta-analysis. BMJ. 2020;371:m4087. 10.1136/bmj.m4087 PMC7610021 33148535

