# Supplementary material for: The projected impact of the COVID-19 lockdown on breast cancer deaths in England due to the cessation of population screening: a national estimation
Source: Br J Cancer. 2022 Feb 2;126(9):1355–61. doi: 10.1038/s41416-022-01714-9 (PMC8808468; doi:10.1038/s41416-022-01714-9)
Supplement: Supplementary file 1 — Supplementary material [file 41416_2022_1714_MOESM1_ESM.docx]

**The projected impact of the COVID-19 lockdown on breast cancer deaths in England due to the cessation of population screening: a national estimation**

**Supplementary Material: Estimating numbers and lengths of delays**

Stephen W. Duffy*, Farah Seedat*, Olive Kearins, Mike Press, Jackie Walton, Jonathan Myles, Daniel Vulkan, Nisha Sharma, Anne Mackie

**Joint first authors*

From national figures and screening activity, it is simple to estimate from previous years and from screening activity in 2020 the numbers of screens which would normally take place, but which have not. A more complex question is the length of the delays in screening. The complex interplay of additional delayed invitations each month, the clearing of the backlog of pre-existing delayed invitations, and the patterns of attendance at screening and lags between invitation and screening include a considerable amount of unknown factors, so some simplifying assumptions were necessary. These included:

1. The number of screens expected under normal circumstances in a single month is uniform. It was calculated as the annual number of screens divided by 12, as 186,210 based on 2018-19 data.(1) Clearly this will be smaller in December and larger in other months, but it is a reasonable working approximation.
2. Invitation and screening activity in any given month will be targeted on the existing backlog, first, with newly eligible persons for invitation in that month having second priority.
3. Monthly screening activity will continue to increase in sigmoid fashion up to a limit, when capacity and attendance reach a steady state.
4. Numbers of delayed invitations by month follow a quadratic curve, that is, they are dependent on both time since the start of the cessation due to the pandemic and on the square of that time. This assumption was arrived at after trying a number of forms for numbers of delayed invitations over time.

We had data on number of screens carried out monthly from April to September 2020. We fitted a sigmoid regression curve of number of screens on calendar month and extrapolated this to estimate screening activity in future months. The observed numbers of screens from April to September 2020 and the estimated numbers thereafter are shown in Table A1. The regression curve reached a limit of 142,348 per month (94% of the figure for 2018-19) for June 2021, and indeed the estimated screening activity was approximately constant for March 2021 onwards. While we cannot know for certain, the limiting number of screens per month of 142,348 seems reasonable, as we might anticipate that screening activity will eventually settle to a figure below that prevailing prior to the pandemic.

Table A2 shows the total number of outstanding delayed invitations at approximately the end of each month (the figures are reported strictly fortnightly and we have taken the dates closest to the end of each month) from the end of May to the end of November 2020. Thereafter the table shows the projected numbers of delayed invitations from the quadratic regression model. The backlog builds up rapidly but starts to reduce from the end of September.

The third column in Table A2 shows the estimated number of screens delayed, assuming that 71.1% of invitations would have resulted in a screen.(1) This will be an underestimate, particularly for later months, as there will be an accumulation of delayed or missed screens in addition to those related specifically to the current number of delayed invitations for any given month, due to the complex relationship of invitation, attendance and time between the two noted above. However, the numbers will be approximately correct for the earlier months and they can be used to estimate how delayed were the screens which actually took place.

We have observed numbers of delayed invitations for May-November 2020, and we fitted a quadratic regression of these on calendar month. We then extrapolated the regression curve to estimate numbers from December 2020 onwards. The extrapolation indicates that the backlog of delayed invitations will be cleared by May 2021, around the same time as the project number of screens per month is predicted to stabilise. Again, note that although the estimated number of delayed screens (71% of delayed invitations) in relation to these delayed invitations reaches zero at the same time as the delayed invitations, this is not the total number of delayed invitations for reasons referred to above.

We can estimate the length of the delay as follows. The hiatus began in mid-March. From Table A1 it can be seen that negligible screening activity took place in April, May and June. Further, 95% (209,638/220,046) of the 220,046 delayed screens at the end of June were already delayed at the end of May. We therefore assume that the average delay for the outstanding screens at the end of June was 2.25 months. With 33,547 screens taking place in July, we assume first that these will be applied to those already in the backlog, so these screens are estimated to have been delayed on average by 2.25 + 0.5 = 2.75 months (i.e. assuming the screens take place uniformly across time within the month). For those estimated to be delayed at the end of the month, we calculate that the accumulated delay is 3.25 months (2.25+1) in 220,046-33,547 = 186,499 women, i.e. the previous backlog minus the screens taking place in July.

The remaining delays, 426,397-186499 = 239898 require another assumption to estimate the delay. Since in a ‘normal’ month, we would expect 186,210 screens, we can assume this number to be due in July and to have a delay of 0.5 months at the end of July. The other 239898 -151,453 = 88,445 are therefore likely to be a legacy from previous months. We assume in the absence of other information that these have a delay of 1.5 months, that is, that they are a legacy only from the previous month.

The overall average delay in months, therefore, in those 426,397 delayed screens at the end of July is therefore estimated as

$$D=\frac{186499\times3.25+186210\times0.5+53688\times1.5}{426397}=1.83$$

For the 73,123 screens taking place in August, we estimate that these were delayed by 2.33 months (1.83 +0.5), again assuming screens take place uniformly over the month). Similar arithmetic applies to calculate the average delays of those screens taking place in subsequent months, and the average delays in those still delayed at the end of each subsequent month. These are shown in Table A3. We estimate that the total number of screens in the year from July 2020 to June 2021 will be 1,489,237, and that the average delay of those screens will be 4.50 months.

This does not represent the entire delay, however. In a ‘normal’ year we would expect 2,234,514 screens. Thus, we estimate that there are an additional 745,277 screens outstanding from the year under consideration. We assume that this population should have been screened uniformly between mid-March 2020 and the end of June 2021. We further assume that this backlog will be screened within twelve months of June 30^th^ 2021, which implies an average delay of 13.25 months (maintaining our assumption of a uniform distribution of screens over time).

| **Table A1. Screening activity recorded for April-September 2020, and extrapolated from these using sigmoid regression for October 2020-July 2021** | | |
| --- | --- | --- |
| **Source of figures** | **Month** | **Number of screens** |
| **Observed data** | April 2020 | 1,638 |
|  | May 2020 | 694 |
|  | June 2020 | 3,472 |
|  | July 2020 | 33,547 |
|  | August 2020 | 73,123 |
|  | September 2020 | 114,277 |
| **Estimated from sigmoid regression** | October 2020 | 133,199 |
|  | November 2020 | 139,690 |
|  | December 2020 | 141,601 |
|  | January 2021 | 142,140 |
|  | February 2021 | 142,290 |
|  | March 2021 | 142,332 |
|  | April 2021 | 142,344 |
|  | May 2021 | 142,347 |
|  | June 2021 | 142,348 |
|  | July 2021 | 142,348 |

| **Table A2. Number of delayed invitations prevalent at the end of each month** | | | |
| --- | --- | --- | --- |
| **Source of numbers** | **Month end** | **Delayed invitations** | **Delayed screens** |
| **Observed numbers** | May 2020 | 294,850 | 209,638 |
|  | June 2020 | 309,488 | 220,046 |
|  | July 2020 | 599,714 | 426,397 |
|  | August 2020 | 950,748 | 675,982 |
|  | September 2020 | 1,260,573 | 896,267 |
|  | October 2020 | 1,171,707 | 833,084 |
|  | November 2020 | 1,026,242 | 729,658 |
| **Extrapolated from observed numbers using quadratic regression** | December 2020 | 1,127,180 | 801425 |
|  | January 2021 | 1,059,200 | 753091 |
|  | February 2021 | 925068 | 657723 |
|  | March 2021 | 724783 | 515321 |
|  | April 2021 | 458347 | 325885 |
|  | May 2021 | 125758 | 89414 |
|  | June 2021 | 0 | 0 |

| **Table A3. Estimated delays for screens taking place July 2020-June 2021** | | | |
| --- | --- | --- | --- |
| **Month** | **Screens in that month** | **Average delay (months) of screens in that month** | **Average delay (months) of backlog at month end** |
| July 2020 | 33,547 | 2.75 | 1.83 |
| August 2020 | 73,123 | 2.33 | 1.92 |
| September 2020 | 114,277 | 2.42 | 2.18 |
| October 2020 | 133,199 | 2.68 | 2.95 |
| November 2020 | 139,690 | 3.45 | 3.78 |
| December 2020 | 141,601 | 4.28 | 3.67 |
| January 2021 | 142,140 | 4.17 | 4.15 |
| February 2021 | 142,290 | 4.65 | 4.81 |
| March 2021 | 142,332 | 5.31 | 5.81 |
| April 2021 | 142,344 | 6.31 | 6.81 |
| May 2021 | 142,347 | 7.31 | 7.81 |
| June 2021 | 142,348 | 5.41 | -* |

*Delayed invitations and therefore delayed screens estimated as zero at end of June

**References**

1. Screening and Immunisations Team, NHS Digital. Breast Screening Programme: England 2018-19. Leeds: NHS Digital; 2020.
